# Organizational factors and depression management in community-based primary care settings

**DOI:** 10.1186/1748-5908-4-84

**Published:** 2009-12-31

**Authors:** Edward P Post, Amy M Kilbourne, Robert W Bremer, Francis X Solano, Harold Alan Pincus, Charles F Reynolds

**Affiliations:** 1 Department of Internal Medicine, University of Michigan, Ann Arbor, Michigan, USA; 2 National VA Serious Mental Illness Treatment Research and Evaluation Center, Ann Arbor Veterans Affairs Medical Center, Ann Arbor, Michigan, USA; 3 Center for Clinical Management Research, Ann Arbor Veterans Affairs Medical Center, Ann Arbor, Michigan, USA; 4 Department of Psychiatry, University of Michigan, Ann Arbor, Michigan, USA; 5 Department of Psychiatry, University of Colorado Medical School, Denver, Colorado, USA; 6 Community Medicine Inc and Center for Quality Improvement and Innovation, University of Pittsburgh Medical Center, Pittsburgh, Pennsylvania, USA; 7 RAND-University of Pittsburgh Health Institute, Pittsburgh, Pennsylvania, USA; 8 Department of Psychiatry, Columbia University, New York, New York, USA; 9 Department of Psychiatry, University of Pittsburgh, Pittsburgh, Pennsylvania, USA; 10 Departments of Neurology and Neuroscience, University of Pittsburgh, Pittsburgh, Pennsylvania, USA

## Abstract

**Background:**

Evidence-based quality improvement models for depression have not been fully implemented in routine primary care settings. To date, few studies have examined the organizational factors associated with depression management in real-world primary care practice. To successfully implement quality improvement models for depression, there must be a better understanding of the relevant organizational structure and processes of the primary care setting. The objective of this study is to describe these organizational features of routine primary care practice, and the organization of depression care, using survey questions derived from an evidence-based framework.

**Methods:**

We used this framework to implement a survey of 27 practices comprised of 49 unique offices within a large primary care practice network in western Pennsylvania. Survey questions addressed practice structure (*e.g*., human resources, leadership, information technology (IT) infrastructure, and external incentives) and process features (*e.g*., staff performance, degree of integrated depression care, and IT performance).

**Results:**

The results of our survey demonstrated substantial variation across the practice network of organizational factors pertinent to implementation of evidence-based depression management. Notably, quality improvement capability and IT infrastructure were widespread, but specific application to depression care differed between practices, as did coordination and communication tasks surrounding depression treatment.

**Conclusions:**

The primary care practices in the network that we surveyed are at differing stages in their organization and implementation of evidence-based depression management. Practical surveys such as this may serve to better direct implementation of these quality improvement strategies for depression by improving understanding of the organizational barriers and facilitators that exist within both practices and practice networks. In addition, survey information can inform efforts of individual primary care practices in customizing intervention strategies to improve depression management.

## Background

Recent reports from the Institute of Medicine suggest that substantial gaps remain between evidence-based care and actual practice [1-3]. This is especially true for chronic conditions. The reports attribute these gaps to organizational barriers in the delivery of longitudinal care and stress the need for future research to identify and reduce barriers to quality and equitable health care. A central challenge is that primary care practices are arranged largely to provide acute treatment; this creates a barrier to improving long-term management of conditions such as depression [4,5].

A body of evidence suggests that, independent of variations in financing, primary care practices differ substantially in how longitudinal care is organized. The effects of environment, ownership, resources, and business management may affect quality of care [6-9]. However, few studies have undertaken to describe organizational factors associated with depression management in primary care settings. Such work is a necessary prerequisite to understanding how organizational factors facilitate or impede treatment and outcomes for depressed primary care patients [10]. Similarly, efforts to implement sustainable evidence-based quality improvement (QI) strategies for depression cannot occur without an understanding of the relevant organizational contexts within primary care practices [11]. This is true because heterogeneity in organizational factors can lead to variation in fidelity to the QI model, ultimately dampening its intended effects.

### Depression highlights the importance of organizational factors in longitudinal care

Depression is one of the most common conditions addressed in primary care [12], and is second only to ischemic heart disease in causing major disability in developed countries [13]. Most Americans receive depression treatment from their primary care physicians (PCPs) rather than mental health specialists (MHS), and thus it is essential that QI efforts occur in this setting [14].

Organizational barriers to longitudinal care in primary care settings are especially detrimental to patients in need of depression treatment [15,16]. Depression remains under-diagnosed and under-treated in primary care practice [15]. Efforts to increase PCP knowledge of appropriate depression treatment and to provide tools for detecting depressed patients have resulted in minimal impact on outcomes. Efforts at improved case recognition are necessary but have not proven sufficient to improve depression management without accompanying efforts that involve organizational change to foster longitudinal care (*i.e*., optimal acute and maintenance treatment) [17].

### A brief history of interventions to improve longitudinal depression management

QI models focused on longitudinal treatment in primary care settings have been developed, notably the chronic care model (CCM) [10,18]. The CCM is designed to facilitate the delivery of longitudinal care through an integrated team composed of different types of providers, often catalyzed by a physician extender (*e.g*., a nurse or a care manager) who promotes patient self-management and systematic use of clinical data and practice guidelines [19]. While not specific to mental health care, this model has been widely applied to depression management interventions, and shown to improve both quality of care and patient outcomes for depression in randomized controlled trials [18,20-24].

However, to date these interventions have not been sustained once the initial grant funding ceased [17,19,25,26]. They were not sustainable in part because they were not adapted to address the fundamental barriers intrinsic to existing organizational structure and processes in primary care practices. Rather, the bulk of the resources and organizational changes to improve longitudinal depression management were implemented through the intervention trial design, and within the time-limited team of study personnel, such that long-term sustainability was unlikely to occur within the practice [25].

Hence, there is a need to identify the organizational barriers and facilitators of depression management, especially within community-based health care settings. To date, many attempts to implement depression management beyond the clinical trial stage have been within health care systems with a central management structure, such as staff-model health plans and Veterans Health Administration (VHA) facilities [27]. These systems can more readily facilitate the diffusion of practice innovations and potentially address the issue of sustainability. However, most Americans receive care within network-model health plans where care is not tightly coordinated, and specialty mental health services are contracted out in the form of carve-out arrangements [28,29]. Network-model plans contract with multiple provider organizations for general medical, behavioral health, and pharmacy benefits. Practices in these organizations are less likely to have incentives or infrastructure to develop systems for longitudinal depression care delivery systems founded on principles from these evidence-based interventions.

Thus, proven interventions for improving longitudinal depression care lack an intrinsic framework to foster sustainability. Consequently, a better understanding of the organizational factors associated with depression management in typical, network-model primary care practices is warranted in order to facilitate sustainable implementation of these interventions [25]. Models of implementing practice change have been developed and applied to similar efforts to improve quality of care for other conditions, notably total quality management [30] and other practice change models [6]. Nonetheless, an explicit framework is necessary in considering how these principles apply specifically to depression management, both in terms of constructing measures of organizational characteristics and in understanding the organizational factors that influence what strategies work best in a given setting [11]. Simply put, the implementation of QI strategies for depression management in primary care cannot progress without a full understanding of 'usual depression care' in network-model settings and key organizational factors associated with longitudinal depression management.

### Purpose of study

The purpose of this study is to describe a framework for characterizing the organizational factors of primary care practice relevant to depression management, and to use this framework in undertaking a survey of network-model primary care practices around longitudinal depression care. Thus, the survey is rooted in an understanding of organizational theory and QI, applied specifically to the structure and processes of depression treatment. Practices within the network where the survey was conducted had variable exposure to time-limited efforts to improve depression care quality. There was no *a priori *expectation that these practices were advanced in their implementation of such efforts. Thus, findings from this study highlight the barriers to longitudinal care for depression in a sample of typical primary care practices, and can inform efforts to advance knowledge of primary care organization and sustainable implementation strategies in the area of depression QI.

## Methods

We describe below the rationale for a quantitative study of the organization of depression management in primary care, development of a conceptual framework to inform a primary care office survey, and the methods by which the survey was implemented within a representative network-model physician organization.

### Depression management in primary care offices

A body of research exists in which attributes of health care organization are characterized across multiple levels. These levels include: patient; provider [31]; practice team or office (distinguished from provider level as it includes other front-line staff); medical group/physician organization [32]; health plan [33]; purchaser; and population/environment levels [34,35]. However, individuals are most likely to identify with their primary care office as their source of care rather than a medical group, health plan, or purchaser, and to perceive their care through interactions with primary care office staff [36].

The primary care office level, while representing the key point of patient contact, has been the least studied [8,37], and there has been a dearth of research characterizing organizational and system-level factors of office staff (*e.g*., to what extent they use information system tools in managing treatment, or identify financial incentives to improve care). A growing body of qualitative research characterizes the diversity and complexity of primary care offices, in particular by combining multifaceted data collection techniques such as direct observation, interviews, and extensive documentation of relationships across different office personnel [38]. However, there has been little quantitative evaluation of office-level organizational features [39-41], particularly with respect to depression care. Studying office-level organization also minimizes the potential for ecologic fallacy; that is, an assumption that relationships between variables at a global level are also present at a lower level of aggregation. This concern is most important in studying higher-level (*e.g*., plan or purchaser level) system attributes, although even at the office level there is unmeasured variation at the provider level.

We also chose a quantitative study approach because it can provide a contextual overview of the impact of office organization on patient-level care. Alternatively, while qualitative data collection can provide in-depth information on organizational processes, it may take extensive time to code and summarize qualitative data to a point where the study may become irrelevant or outdated for use in implementation. Moreover, qualitative data are more suitable for hypothesis generation, while quantitative data on organizational factors can be used to test specific hypotheses regarding the relationship between structure, processes, and outcomes of depression management. Hence, changes to the organization of care at the office level that are informed by quantitative studies can have a more immediate impact on patient-level processes and outcomes [36].

### Conceptual framework development

**Figure 1:**
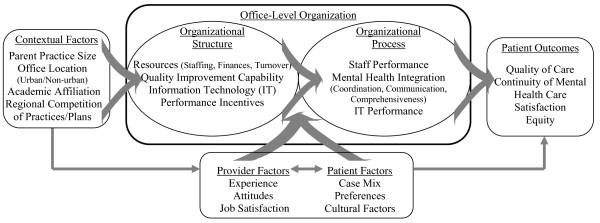
Conceptual framework of depression care organization

To guide the establishment of a quantitative survey to address depression care organization, we developed a framework that describes the underlying concepts of primary care organization as a practical means of benchmarking the structure and process of depression management. The framework for our organizational survey characterizes the key barriers and facilitators of good depression treatment in routine primary care practice and is illustrated in Figure 1. It draws concepts from several sources, and assembles these concepts into a framework in a manner that is informed by experience in both clinical management and effectiveness research. One source is the health services organizational research by Zinn and Mor [42] and Shortell and colleagues [43,44], among others. This work includes the concept that patient-level processes and outcomes of care are influenced by underlying characteristics of the health care environment. Our framework proposes that the organizational structure of the office influences the processes by which depression treatment is delivered and ultimately impacts patient-level outcomes [42,45]. A second source that influenced our approach for characterizing organizational factors is the Donabedian quality framework, which describes how health care structure (*e.g*., resources) can influence quality of care at the patient level and subsequent outcomes [45]. Similar to Donabedian, our framework also defines patient outcomes broadly to include processes and outcomes of treatment as well as measures of equitable care and patient acceptance of care [2,46,47]. Additional domains outlined in this framework are not the immediate focus of our survey, but include underlying provider and patient factors. Provider factors, including experience, attitudes regarding QI in general and depression in particular, and job satisfaction, can influence patient outcomes [31,48,49]. Patient factors influence the decision to seek treatment and affect subsequent outcomes. These include depression severity, cultural and sociological factors, and treatment preferences [50]. With its emphasis on clinical management, our framework emphasizes the centrality of structural elements as a prerequisite to many processes. This distinguishes it from the Promoting Action on Research Implementation in Health Services (PARIHS) framework [51], which relies on a social psychology approach in delineating the presence of evidence, context/culture, and facilitation as factors that increase the probability of successful implementation.

### Organizational survey

The primary care depression management organizational survey was developed based on our conceptual framework, which includes four major domains: contextual factors, organizational structure, organizational processes, and patient outcomes (Figure 1). Organizational structure features are defined as factors related to staffing or capital/financial resources within the office, human resource factors, information technology (IT) infrastructure, financial measures, and QI expertise. Organizational processes refer to the management and specific use of resources, such as IT and the degree to which elements of mental health are integrated into primary care practice. Contextual factors are defined as the factors external to the office that may influence the office's organization or delivery of care. Patient-level outcomes include quality of care, satisfaction, and other factors thought to be directly influenced by organizational characteristics [45].

Survey questions were initially selected based on empirical studies that addressed the relationships between these domains. These studies focused on either depression management, or upon other chronic illnesses that share common features of depression management, such as longitudinal care and coordination between different provider specialties (*e.g*., mental health, primary care providers) [9,31,32,42,43,52-58].

Based on this review, we then selected questions previously used in other studies to fit within each domain and conducted a careful analysis of empirical studies of primary care and mental health organization. We focused on identifying questions that were not only important correlates of improved depression management, but were also measurable and potentially mutable. As part of this step, we assessed published measures and contacted experts and colleagues to evaluate unpublished measures of organizational features and recommend measures based on their importance, measurability, and mutability. The questions, derived from prior organizational studies, are summarized in Table 1 and operationalize the constructs contained in each domain for use in our survey. The survey instrument is presented in an additional file 1.

**Table 1:** Depression care organizational survey elements.

Framework domain	Key variables^a^	Responses	Reference^b^
Organizational structure			

Resources			

Staffing	Staffing volume and mix	Total # of staff; Ratio of (NP+PA) to MDs	Yano 2000 [9]

Finances	Financial stress	Worry about finances a little or a lot; No worry	Meredith 1999 [31]

Turnover	Proportion of staff who were not working in office 2 years ago	%	Rost 2001 [55]

Quality improvement capability	Office ever implemented a quality improvement program for a chronic condition	Yes; No; Don't know	Casalino 2003 [32]

	Clinical reminders for depression care	Yes; No; Don't know	Casalino 2003 [32]

	Formal screening method for depression	Yes; No; Don't know	Casalino 2003 [32]

Information technology infrastructure	Use of electronic medical record	Yes; No	Casalino 2003 [32]

	Registry for depressed patients	Yes; No	Casalino 2003 [32]

Performance incentives	Types of financial and non-financial incentives used in general and for depression care	Quality or Productivity bonus; Compensation at risk; Publicizing performance; Insurance	Casalino 2003 [32]

Organizational process			

Staff performance	How often do providers in office regularly meet	Weekly; Biweekly; Monthly;	Rost 2001 [55]
		Quarterly; No regular meetings	

Mental health integration			

Coordination	Access to mental health specialist	Yes: < 4 blocks; Yes: > 4 blocks; No	Yano 2000 [9]

	Primary locus of depression care for patients without comorbidities; with substance use disorder; with psychiatric comorbidities; and with major medical comorbidities		Yano 2000 [9]

	Diagnostic, CPT codes used for depression diagnosis and treatment	Depression-related; Non-depression related; Total time	Rost 1994 [56]

	Difficulty in arranging an appointment for patients with a mental health specialist (MHS)	Never; Rarely; Sometimes; Often; Always	Yano 2000 [9]

Communication	Typical mode of communication	No communication;	Morrissey and Burns
		Yes (e.g., by telephone, letter, referral form)	1990 [57]; Shortell 1991 [43]

	How often PCP communicates with MHS	Never; Rarely; Sometimes; Often; Always	Miles 2003 [58]

	Does PCP hear whether patient made MH appt	Yes; No	Miles 2003 [58]

Comprehensiveness	Presence of psychologist, psychiatrist, psychiatric social worker, psychiatric nurse, or other mental health specialist in office	Any MHS; None	Yano 2000 [9]

	Case management for depression	Yes; No	Yano 2000 [9]

Information technology performance	Information technology implementation scale	Summary score	Doebbeling 2004 [52]

Contextual factors			

	Practice size	# Offices in practice	Casalino 2003 [32]

	Office location (urban, non-urban)	Urban: in Pittsburgh; Suburban: outside Pittsburgh	Yano 2000 [9]

	Academic affiliation (i.e., office involved in resident or medical school teaching)	Yes; No	Yano 2000 [9]

^a^Variables are included if they are: important (to primary care organization or patient care), measurable, and mutable (able to be modified at the primary care office level).

^b^Includes references for measures that have been applied to primary care settings directly or can potentially be derived for use in primary care settings.

Given the focus on primary care, many questions were derived from the VHA Primary Care Practices Survey [9]. Designed to provide a foundation for evaluating organizational structure and processes, its content was built on similar theoretical models to those we used in our framework [42-44]. The Primary Care Practices Survey was validated using an expert panel integrating nominal group techniques for achieving consensus [59,60]. The process emphasized integration of evidence from published literature with expert opinion to arrive at organizational measures hypothesized to influence key outcomes, including quality of care and patient satisfaction. Structured interviews of facility directors, chiefs of staff, front-line providers, staff, and patients were conducted to validate selected constructs. The resulting constructs were translated into questionnaire items using standard techniques, pilot tested among primary care leaders from diverse practice settings to ensure reliability, and refined iteratively in arriving at a final instrument.

We derived additional variables from studies listed in Table 1. Given the experiences of prior investigations [9], we did not consider 'subjective' questions regarding integrated care (*e.g*., attitudes or perceived effectiveness). These questions could lead to response bias, such as selective nonresponse or affirmative responses about the success of treatment protocols [61]. We outline below the survey variables within the domains of organizational structure, organizational process, and contextual factors.

### Survey measures: organizational structure

Organizational structure consists of the following elements related to human resources, capital assets, or financial measures: staffing, QI capability, IT infrastructure, and external performance incentives.

The domain of resources includes questions on staffing volume and mix [9], financial health, and turnover. Evidence suggests that primary care-based nurse practitioners (NPs) and physician assistants (PAs) may be more likely than physicians to deliver preventative care [62] and mental health/substance use care [63].

An emphasis on QI capability is an important component of organizational structure [43,64,65]. For example, experience with QI programs in VHA clinics [9,40] and by physician organizations has been linked to increased use of longitudinal care management processes [32]. Formal screening and use of clinical reminders was also associated with a greater probability of ongoing care for depression [32].

IT infrastructure includes the availability of an electronic medical record (EMR), and is useful for the long-term follow-up required for chronic illnesses [32]. The presence of this infrastructure can gauge a clinic's readiness to implement depression care management. Casalino and colleagues [32] found that physician organizations with more sophisticated IT--defined as the ability to generate problem lists, real-time progress notes, medication lists, and ordering reminders and/or drug-drug interaction information--were more likely to deliver care consistent with the CCM.

External performance incentives, often arising from health plans or physician organizations, can influence the capacity for delivering longitudinal care [32]. External incentives include financial as well as non-financial incentives that are used to improve quality or curb costs.

### Survey measures: organizational process

Three key domains referable to the management and specific use of resources define organizational process: staff performance, degree of mental health integration, and IT performance.

Staff performance includes teamwork [66], defined as communication and problem solving among staff to ensure that expertise is available to solve problems [43]. Multiple studies have shown that a high degree of teamwork was associated with improved quality of process and outcomes in primary care and other settings [64,67,68].

Integrated care is also an important component of our framework [69,70] and contains several subdomains: coordination, communication, and comprehensiveness [57,58,71]. Coordination is defined as the degree to which PCPs and MHSs establish linkages with each other [57] and use common procedures (such as explicit coding of mental health diagnoses) in the process of delivering depression care [56,71]. In the context of primary care, the key coordination variables are MHS location, difficulty in arranging specialist referrals, and coding/billing practices. Shortell and colleagues [43] found that a high degree of services coordination between specialties was associated with improved quality and outcomes in intensive care units. Communication is defined as the degree that patient treatment information is shared by PCPs and MHSs, as well as the use of common protocols to share this information [43,57,58,72]. Comprehensiveness [73] is the extent to which depression care is provided on-site [63].

IT performance is assessed using the Information Technology Implementation Scale [52,74]. More sophisticated adoption of IT, independent of IT infrastructure, has been linked to better coordination of longitudinal care and QI [75]. Doebbeling and colleagues [52] derived dimensions of IT recommended in the Institute of Medicine's report 'Crossing the Quality Chasm'. The scale measures five dimensions of IT implementation using a five-point Likert scale: computerized clinical data, electronic communication between providers, automation of decisions to reduce errors, access to literature/evidence-based medicine while delivering care, and decision support systems. We summed numerical responses to these items in deriving an 'IT implementation' score that can range from zero to 20.

### Survey measures: contextual factors

Contextual factors include measures of practice size (number of office locations), urban/non-urban location, and academic affiliation from the Primary Care Practices Survey [9]. All of these factors were found to be associated with depression care referral practices [63].

### Conducting the survey: study design and analysis

We conducted a cross-sectional study of primary care offices within Community Medicine, Inc., which is a large network-model physician organization located in Allegheny County, Pennsylvania. This area includes Pittsburgh and many of its surrounding suburban communities. Network-model physician organizations are typically large groups of individual offices or practices. We identified offices from the network list of unique facilities, excluding offices that provided only specialty care. Within the network-model organization, some offices were organized into groups called 'practices'. An office is defined as a stand-alone building or clinic, while a practice is defined by a group of offices under the same local management team, with at least partial overlap of providers between offices.

The practice manager served as the primary respondent to survey questions recorded for each unique office location within the practice. Surveys were administered in-person by a trained research assistant, and the survey took approximately 30 minutes to complete. We asked that the practice manager refer to a clinical designee for any questions beyond the scope of their knowledge. This use of key informants to ascertain characteristics of a site is a well-established practice in organizational research. Key informants interact directly with patients and staff as well as practice and plan representatives, and thus are considered the most knowledgeable about the delivery of care at the office and the policies regarding specialty services external to the practice. This approach helps to provide a comprehensive picture of primary care organization. The study protocol was reviewed by the University of Pittsburgh Institutional Review Board (reference number 0411077), and designated as exempt: informed consent from respondents was not required since the data collected related to the characteristics of primary care offices.

In analyzing our results, we used descriptive statistics to report the survey measures; namely, means, medians, and standard deviations for continuous variables and frequencies for categorical variables. Because some offices were clustered under a single practice, results were reported by practice for responses that reflect factors that are constant across office locations within practices (*e.g*., external incentives) or reflect shared resources across locations (*i.e*., staff). We performed analysis using SAS Version 8.2 (SAS Institute, Cary, NC).

## Results

The survey was completed by 27 of 30 (90.0%) eligible primary care practices representing 49 out of 53 (92.5%) office locations within the network.

### Sample description and contextual factors

The practice sample is described in Table 2. All offices provided adult care, while approximately one-half provided care to adolescents and one-quarter to children. The 27 practices ranged in size from one to five office locations, with a median of two offices. Approximately one-third (36.7%) of these offices were in Pittsburgh, with the remainder in suburban locations. Finally, 73.5% of offices participated in resident or medical school teaching.

**Table 2:** Practice sample and contextual factors.

Factor	Responses	Offices	%
Primary care practices surveyed	*N *= 27 practices		
Unique office locations and populations served	*N *= 49 offices		
	Provide care to:		
	Adults	49/49	100.0
	Children	11/49	22.5
	Adolescents	24/49	49.0
Parent practice size	Median office locations	2	
	Range	1 to 5	
Office location (urban, non-urban)	Urban	18/49	36.7
	Suburban	31/49	63.3
Academic affiliation (i.e., office involved in	Yes	36/49	73.5
resident or medical school teaching)	No	13/49	26.5

### Organizational structure

Structural characteristics of these practices center on the domains of resources (*e.g*., personnel, turnover, financial stress), QI capability, IT, and external performance incentives (Table 3). Personnel were not necessarily exclusive to one office location within a practice; therefore, we calculated staffing statistics per location for each of the 27 practices. The mean number of staff (inclusive of provider and administrative personnel) for each office was 11.8 ± 9.8 persons. Physicians comprised the bulk of the provider staff, with a mean NP and PA:MD ratio of 0.12. Staff turnover was low (6.2%) on average but ranged from zero to 50%. Most practices had little financial stress, with 96.3% reporting no worry or little worry about finances.

**Table 3:** Organizational structure.

Factor		Responses		
Resources				
	Staffing: Volume and mix per office location	(*N *= 27 practices)		
	Total # of persons	Mean ± SD	11.8 ± 9.8	
	Ratio of (NP+PA) to MDs	Mean ± SD	0.12 ± 0.25	
	Turnover: Proportion of practice staff who were not working in office 2 years ago	Mean ± SD	6.2 ± 12.3%	
		Range	0% to 50%	
		No turnover	44.4%	

**Factor**		**Responses**	**Practices**	**%**

Resources				
	Finances: Financial stress	Worry a little	11/27	40.7
		Worry a lot	1/27	3.7
		No worry	15/27	55.6
QI capability				
	Office ever implemented a quality improvement program for chronic condition	Yes	22/27	81.5
		No	5/27	18.5
	Formal screening method for depression	Yes	20/27	74.1
		No	5/27	18.5
		Don't know	2/27	7.4
	Clinical reminders for depression care	Yes	4/27	14.8
		No	22/27	81.5
		Don't know	1/27	3.7
Performance Incentives				
	Types of financial and non-financial incentives used in general and for depression care	Quality bonuses		
		General	10/27	37.0
		Depression	3/27	11.1
		Productivity bonuses		
		General	12/27	44.4
		Depression	2/27	7.4
		Compensation at risk		
		General	5/27	18.5
		Depression	0/27	0.0
		Publicizing performance		
		General	1/27	3.7
		Depression	1/27	3.7
		Insurance incentives		
		General	18/27	66.7
		Depression	5/27	18.5

**Factor**		**Responses**	**Offices**	**%**

Information Technology (by office location)				
	Use of electronic medical record	Yes	17/49 offices	34.7
		No	32/49 offices	65.3
	Registry for depressed patients	Yes	39/49 offices	79.6
		No	10/49 offices	20.4

QI capability among the practices was high, but did not appear to be advanced with respect to depression treatment. A large majority (81.5%) of practices reported implementing QI programs for chronic conditions. Similarly, many practices (74.1%) stated that they employed a formal method of depression screening. However, only four of 27 practices (14.8%) used clinical reminder systems for depression management.

IT infrastructure varied significantly by location within practices, so we report statistics for the 49 office locations in our sample. Many offices (65.3%) were not currently using an EMR. However, a majority of offices (79.6%) reported having a registry for depressed patients.

Finally, external performance incentives were prevalent but less likely to extend specifically to depression care. Each method of incentive (quality bonus, productivity bonus, compensation at risk, publicizing performance, and insurance incentives) existed. However, quality bonuses (37.0% of practices), productivity bonuses (44.4% of practices), and insurance incentives (66.7% of practices) were the most common ways of influencing primary care in general. The use of these methods as a way of improving depression management was much lower, with respective practice prevalences of 11.1%, 7.4%, and 18.5%.

### Organizational process

Factors relating to the organizational process of these practices are delineated in Table 4 across the domains of staff performance, mental health integration, and IT performance. Staff performance was measured by the frequency of provider meetings. Most practices (81.5%) held monthly provider meetings.

**Table 4:** Organizational process.

Factor		Responses	Practices	%
Staff Performance				
	How often do providers in office regularly meet	Weekly	2/27	7.4
		Monthly	22/27	81.5
		Quarterly	3/27	11.1
Mental health integration				
	Coordination			
	Primary locus of depression care	For patients without comorbidities		
		PCP in Office	23/27	85.2
		MHS in PCP Office	0/27	0.0
		Sent to MHS	3/27	11.1
		Don't know	1/27	3.7
		For patients with substance use disorder		
		PCP in Office	14/27	51.9
		MHS in PCP Office	2/27	7.4
		Sent to MHS	10/27	37.0
		Don't know	1/27	3.7
		For patients with psychiatric comorbidities		
		PCP in Office	14/27	51.9
		MHS in PCP Office	0/27	0.0
		Sent to MHS	12/27	44.4
		Don't know	1/27	3.7
		For patients with major medical comorbidities		
		PCP in Office	20/27	74.1
		MHS in PCP Office	0/27	0.0
		Sent to MHS	6/27	22.2
		Don't know	1/27	3.7
	Diagnostic, CPT codes used for depression diagnosis and treatment (multiple codes per practice)	ICD9 Codes		
		Depression-related	27/42	64.3
		Non-depression related	15/42	35.7
		CPT Codes		
		99213 billing code	24/58	41.4
		Median time: 25 minutes		
	Difficulty in arranging an appointment for patients with a mental health specialist (MHS)	Not Applicable	18/27	66.7
		Never	4/9	44.4
		Rarely	1/9	11.1
		Sometimes	1/9	11.1
		Often	1/9	11.1
		Always	2/9	22.2
	Communication			
	Typical mode of communication	No Communication	0/27	0.0
		Yes (various forms)	27/27	100.0
	How often PCP communicates with MHS	Never	0/27	0.0
		Rarely	3/27	11.1
		Sometimes	15/27	55.6
		Often	3/27	11.1
		Always	5/27	18.5
		Don't know	1/27	3.7
	Does PCP hear whether patient made MH appointment (choose all that apply)	Yes		
		PCP Calls	1/27	3.7
		PCP Asks Patient	14/27	51.9
		Other	9/27	33.3
		No	5/27	18.5
		Don't know	1/27	3.7
	Comprehensiveness:			
	Presence of mental health specialist in	Any MHS	2/27	7.4
	PCP office	None	25/27	92.6
	Case management for depression	Yes	7/27	25.9
		No	20/27	74.1

**Factor**		**Responses**	**Offices**	**%**

Mental health integration				
	Coordination			
	Access to mental health specialist	Yes, < 4 blocks	6/49	12.2
		Yes, > 4 blocks	26/49	53.1
		No	17/49	34.7
Information technology performance				
	IT implementation scale (maximum = 20)	(*N *= 49 offices)		
		IT Score Mean ± SD	10.7 ± 4.2	
		Range	3 to 17	

Mental health integration was characterized by measures capturing coordination and comprehensiveness of care, as well as communication. Few offices were able to provide coordinated depression care through co-location of a MHS on-site (8.2% of offices) or within four blocks (12.2% of offices). Similarly, few practices (25.9%) had a depression case management program. However, most provided treatment for uncomplicated depression (85.2% of practices) and depression treatment for medically complicated patients (74.1% of practices). The prevalence of referral to a MHS's office was greater in the presence of substance use (37.0% of practices) and psychiatric comorbidities (44.4% of practices). A majority of practices (66.7%) did not arrange specialist appointments for patients. Among those that did, the greatest number reported never having difficulty in arranging for a specialist appointment although the responses spanned the five-point Likert scale. All practices reported some communication with the MHS. The frequency varied, with many practices (55.6%) reporting communication sometimes; 11.1% communication often; and 18.5% communication always. Despite this ongoing communication regarding depression management, many practices (51.9%) reported that knowledge of the patient making their MHS appointment occurred through PCP inquiry to the patient.

Finally, primary care offices reported an intermediate level of IT implementation on average. Specifically, the mean IT scale score was 10.7 ± 4.2 across the 49 offices, with values ranging from three to 17.

## Discussion

This is one of the first studies to elaborate upon a framework for examining the organizational barriers and facilitators of depression management within community-based primary care offices, and to operationalize and refine a survey for accomplishing this task. Surveying the organizational features of offices is one of the most efficient ways to identify factors associated with successful implementation of improved longitudinal care, and is a prerequisite to finding sustainable solutions to improving depression management.

Understanding the organization of longitudinal depression management at the office level is important, because most QI interventions are targeted to change the processes of care within the office. Identifying organizational features that are mutable is critical to implementing depression treatment interventions. It is also important, in adapting these interventions, to better fit less mutable organizational features. QI initiatives, including the Institute for Healthcare Improvement's Breakthrough Series [10] and the Robert Wood Johnson Foundation's Depression in Primary Care Program [17], have focused on organizational changes at the office level. However, the degree to which their recommendations were implemented varied, and ultimately had to be customized to the individual practice environment. Hence, standardizing a method for collecting data on organizational features, including barriers and facilitators of good depression treatment, can guide implementation efforts greatly. Furthermore, validation of these methods by determining their links to patient outcomes is essential to assure the effective customization of treatment models.

Our study demonstrated substantial variation in the organization and delivery of longitudinal depression care in usual primary care settings. Specifically, the QI capability of the surveyed practices was high but not currently focused on depression management. Clinical reminder systems and case management for depression were unusual. The use of information tools varied widely, as evidenced by variation in uptake of EMRs and the wide range of IT implementation scores. Notably, less than 15% reported using clinical reminder systems specifically for depression, suggesting that the structural capabilities of most practices have not extended to facilitating follow-up and longitudinal depression care activities. However, a majority of offices (79.6%) reported having a 'registry' of depressed patients, despite the fact that far fewer had an EMR. The fact that many offices without EMRs had a registry of depressed patients in place may be explained by the presence of several QI initiatives by the dominant insurers in the region, which have included depression management in primary care. Nonetheless, further research is needed to determine whether 'top-down' implementation of IT tools, such as centrally maintained registries, facilitates or impedes organizational processes that already exist in the office (*e.g*., EMRs).

Coordination and communication, important prerequisites for depression management, also varied significantly, likely a reflection in process of the variation that this survey demonstrated in practice structure. Finally, external incentives for improving depression management were much less prevalent than incentives focused on care in general.

There are limitations to this study that warrant consideration. First, our survey represents an initial attempt to refine and implement an assessment of organizational factors in depression treatment. Still, the offices included within this study are part of a network-model physician organization that represents the typical health care setting for many Americans, because they include a mixture of urban, suburban, and more rural office locations. Additional work is needed using a more diverse national sample of practices and multiple key informants per office location to better characterize the relationship between organizational structure and organizational processes within the context of depression management, particularly within a larger, national sample of primary care practices. Second, we were unable to assess measures of stability of the responses or validity (beyond the face validity based on our framework). In addition, while noted in the conceptual framework, we were not able to assess the impact of organizational features on patient outcomes or determine whether provider or patient factors confounded these relationships. Patient-level outcomes data were not available from practices at the time of the survey. We were also unable to determine whether patient or provider factors influenced the relationship between organizational structure and depression care process. Nonetheless, recent evidence suggests that the majority of variation in patient outcomes was explained by office-level and not provider-level variation [7]. Moreover, many of the organizational features that were assessed, notably organizational processes, are based on key informants' perspectives and may not represent the actions of all providers in the office (*e.g*., not all providers in offices with clinical reminders for depression may act upon the prompts). Nonetheless, information from key informants can be a more efficient way to collect information on a broad array of organizational barriers and facilitators than relying on survey responses from multiple providers. Finally, we were unable to assess other contextual factors such as health plan or practice competition, as this would have required a larger survey of offices across several regions and practice organizations.

## Conclusions

A better understanding of how depression care is organized and delivered in real-world practices can lead to improved depression management by identifying and studying the mutable factors at the office level that impede integrated care. Quantifying organizational barriers and facilitators can also produce a practical needs assessment for practices by examining their organizational structure, and can serve as a marker for their readiness to improve depression management. Further understanding the unique organizational barriers at the primary care office level can help practitioners and researchers customize QI strategies for care of depression by matching depression care management strategies with specific organizational characteristics (*e.g*., IT variation, staffing mix). Additional research is needed to determine which mutable organizational characteristics at the primary care level impact depression-specific outcomes. Moreover, further information on appropriate depression care organization and treatment models for special populations (*e.g*., children, elderly) is needed. Ultimately, further understanding of the organizational barriers and facilitators of depression management in primary care settings is required to develop next-generation depression management models that are adapted to the organizational barriers and hence, likely to be sustainable in network-model primary care practices.

## Competing interests

The authors declare that they have no competing interests.

## Authors' contributions

EPP participated in the design of the study, acquired the data, performed the statistical analysis and drafted the manuscript. AMK conceived of the study and helped to draft the manuscript. RWB participated in the design of the study and helped to draft the manuscript. FXS participated in the study design and acquisition of data. HAP participated in the design of the study. CFR participated in the design and coordination of the study. All authors critically revised the manuscript for important intellectual content, read, and approved the final manuscript.

## Supplementary Material

Additional file 1 **Survey**. Survey instrument used in this study. Click here for file
