# Supplementary material for: Organizational factors and depression management in community-based primary care settings
Source: Implement Sci. 2009 Dec 31;4:84. doi: 10.1186/1748-5908-4-84 (PMC2813228; doi:10.1186/1748-5908-4-84)
Supplement: Additional file 1 — Survey. Survey instrument used in this study. [file 1748-5908-4-84-S1.PDF]

Office ID __ __ __ __ Location: City ________________________ State __ __ Date __ __/__ __/__ __ __ __

PRIMARY CARE SURVEY

A. Organizational Characteristics

We want to learn more about the general features of your office.

A1. What health-related services does your office provide (check all that apply)?
__General medical care __Substance use treatment __Psychiatric care
__Other:____

A2. Does this office include (check all that apply)?
__Children __Adolescents __Adults __Geriatrics __Other
__Specify Other:____

A3. Please estimate how many of the following presently work in your office:

	Total Number of Staff	Total Full-Time Equivalents
MDs	____	____
Physician assistants (PAs)	____	____
Nurse practitioners (NPs)	____	____
Registered nurses (RNs)	____	____
Medical Assistants	____	____
Social workers (MSWs)	____	____
Psychiatrists	____	____
Psychologists	____	____
Other Behavioral Health providers	____	____
Other non-behavioral health staff	____	____

A4. What percentage of individuals working in your office today were not employed in this office 2 years ago?___%

A5. Is your office involved in resident or medical school teaching? ___ Yes ___ No

A6. How many different **office locations** are part of the practice you are in? ________________________________

A7. How would you rate the "financial stress" in your office presently?
___ Not a problem ___ Worry about finances a little ___ Worry about finances a lot

A8. How often does your office staff meet?
__Once per week ___Biweekly __Once per month __Once per quarter __No regular meetings held

A9. Has your office ever participated in any quality assurance (QA) or quality improvement (QI) activities?
___ No ___ Yes (Describe **most recent** program:____

____
____

A10. Please check the types of quality improvement incentives for which your office, or any providers within your office are eligible (check all that apply):

	Used in general	Used for depression care
**a.** Quality bonuses for the office or individual physicians		
**b.** Provider productivity bonuses		
**c.** Compensation at risk		
**d.** Publicizing performance		
**e.** Insurance incentive programs (e.g., UPMC, Highmark)		
**f.** Other (please specify: ____)		

GENERAL NOTES ____
____
____
____
____

Office ID __ __ __ __   Location: City ________________________ State __ __   Date __ __/__ __/__ __ __ __

B. Practice Features .

The following questions address access to medications for patients in your office

B1. How often are providers in your office detailed by a representative from a pharmaceutical company?
__Daily ___ ___ x per ______ ___Once per week ___Biweekly __Once per month __Once per quarter
__Rarely/never

B2. In what ways do you make it easier for patients in your office to obtain medication prescriptions?
(please check all that apply)
__Provide free samples
__Enroll in pharmaceutical company assistance programs
__Charity funds
__Other (specify:____________________________)

B3. Approximately what percentage of your patients in your office obtain free samples of **medications** from pharmaceutical companies?
Once at the time of diagnosis ____%
On an ongoing basis ____%

The following questions ask about the clinical information systems typically used in your office

B1. How many computers do you have in this office location? __ __ __ computers

B2. What computerized billing software program is used in your office?
__ EPIC® __Other program (Specify__________________________________) __No computerized billing program used

B3. What computerized appointment scheduling program is used in your office?
__EPIC® __Other program (Specify__________________________________) __No computerized program used

B5. What electronic medical record program is used in your office?
__CERNER® __Other program (Specify__________________________________) __No electronic program used

**B6.** Indicate to what extent your facility provides information technology for:	NOT AT ALL	VERY LITTLE	SOME	GREAT	VERY GREAT
a. Access to literature/evidence based medicine while delivering care	1	2	3	4	5
b. Computer assisted decision support systems (e.g., condition-specific lab tests or medications)	1	2	3	4	5
c. Computerized patient clinical data (e.g., problem list, history of adverse drug reactions, or electronic medical records)	1	2	3	4	5
d. Automation of decisions to reduce errors (e.g., potential adverse drug interactions, correct dosages)	1	2	3	4	5
e. Electronic communication between providers (e.g., e-mail, automated result reporting)	1	2	3	4	5
f. Electronic communication between providers and patients (e.g., e-mail, automated result reporting)	1	2	3	4	5
g. Clinical reminders generated by a computer tracking system	1	2	3	4	5

GENERAL NOTES ____
____
____
____

C. Chronic Illness and Preventive Care Functioning and Information

C1. To what extent does your office use case managers or other non-physician staff to help physicians take care of patients with asthma, congestive heart failure (CHF), depression, or diabetes? (CHECK ALL THAT APPLY)

	Asthma	CHF	Depression	Diabetes
**a.** The office does not use case managers for this condition (SKIP C**1b** AND C**1c, GO TO** C**2**)				
**b.** Case managers are available at the request of the physician				
**c.** Case managers are assigned to all severe cases				

C2. Does your office maintain a registry/or list of its patients with the following conditions?

	Yes	No
**a.** Asthma	___	___
**b.** Congestive heart failure	___	___
**c.** Depression	___	___
**d.** Diabetes	___	___

C3. For which of the following does your office contact patients for purposes of improving compliance with recommended screening or treatment protocols?

	Yes	No	N/A
**a.** For patients over the age of 50, does your office send reminders to patients regarding colorectal cancer screening?	___	___	___
**b.** For children, does your office send reminders to parents regarding immunizations or well child visits?	___	___	___
**c.** For persons with diabetes, does your office send reminders to patients regarding eye exams?	___	___	___
**d.** For women, does your office send reminders to patients regarding osteoporosis screening?	___	___	___
**E.** For men, does your office send reminders to patients regarding prostrate cancer screening?	___	___	___

D. Depression Management in Primary Care

The following questions ask about how depression care is typically delivered by the GENERAL MEDICAL providers in your office

D1. Do providers in your office use guidelines or protocols to care for patients with depressive disorders?
___ No (**SKIP** to **D2**) ___Yes →

D1a. (If **YES** to D**1**) Please specify which of the following are covered by your office's guidelines or protocols for the management of depression:
___ Use of referrals
___ Use of antidepressants
___ Screening for depression
___ Monitoring treatment response and follow-up

D2. Do providers in your office use a formal method of screening for depression?
___ No screening tool or questionnaire is used at our office (if **NO**, **SKIP** to **D3**)
___ Don't Know
___ Yes, a self-report questionnaire →
___ Yes, a standardized instrument is administered by a nurse →
___ Yes, Other method →

D2a. (If **YES** to **D2**) Please name the most common method used to screen for depression:_________________
____
____

GENERAL NOTES ____
____
____

Office ID __ __ __ __ Location: City ________________________ State __ __ Date __ __/__ __/__ __ __ __

D3. What diagnostic codes (ICD-9 codes) do the providers in your office typically use to bill for depression care?

_________ _________ _________ ________

D4. What billing codes (CPT codes) do the providers in your office typically use to bill for depression care?*

_________ _________ _________ ________

D5. How are patients with depressive disorders **primarily** cared for? (Select **one** number for each level of severity)

	**a.** Depression with no comorbidity	**b.** Depression with coexisting alcohol or drug problem	**c.** Depression with coexisting psychiatric diagnosis	**d.** Depression with coexisting major medical comorbidity
Patients are primarily cared for by general medical providers in the office	1	1	1	1
Patients are primarily cared for by psychiatrists or other mental health specialists **in your office**	2	2	2	2
Patients are primarily **sent** to specialized programs **outside the office**	3	3	3	3

The following questions ask about how referrals are typically handled by GENERAL MEDICAL providers in your office

D6. How would you rate the difficulty in arranging an appointment for one of your patients with any **MEDICAL specialist** (e.g., cardiologist, endocrinologist, oncologist)?

___ Never a problem **(SKIP to D7)**
___ Rarely a problem →
___ Sometimes a problem →
___ Often a problem →
___ Always a problem →

D6a. Please rank the reasons why providers in your office experience difficulty in arranging an appointment for one of your patients with any **MEDICAL specialist**:

___ Unaware of who providers are
___ Preferred specialist provider is unavailable in my office's network
___ Shortage of specialists
___ Other (please specify: ____)

D7. How would you rate the difficulty in arranging an appointment for one of your patients with any **MENTAL HEALTH specialist** (e.g., psychiatrist, psychologist, etc.)?

___ Never a problem **(SKIP to D8)** ___ **N/A (SKIP to D8)**
___ Rarely a problem →
___ Sometimes a problem →
___ Often a problem →
___ Always a problem →

D7a. Please rank the reasons why providers in your office experience difficulty in arranging an appointment for one of your patients with any **MENTAL HEALTH specialist**:

___ Unaware of who mental health providers are
___ Have to go through a mental health carve-out
___ Preferred mental health provider is unavailable in my office's network
___ Shortage of mental health providers
___ Other (please specify: ____)

GENERAL NOTES ____

D8. How would you rate the difficulty in arranging an appointment for one of your patients with any **SUBSTANCE USE DISORDER specialist** (e.g., addictions specialist, etc.)?

___ Never a problem **(SKIP to D9)**   ___ **N/A (SKIP to D9)**
___ Rarely a problem →
___ Sometimes a problem →
___ Often a problem →
___ Always a problem →

D8a. Please rank the reasons why providers in your office experience difficulty in arranging an appointment for one of your patients with any **SUBSTANCE USE DISORDER specialist**:

___ Unaware of who substance use disorder providers are
___ Have to go through a mental health carve-out
___ Preferred substance use disorder provider is unavailable in my office's network
___ Shortage of substance use disorder providers
___ Other (please specify: ______________________________)

D9. Do you have access to a **specific Mental Health Specialist** or a group of Mental Health Specialist providers (e.g., psychiatrist, psychologist, or other mental health specialist) who provide mental health and/or substance abuse treatment for patients who visit your office (regardless of whether he or she is an employee of the office)?

___ Yes, located on site-in the office → **SKIP to D11**   ___**Don't Know**
___ Yes, located off site-less than 4 blocks away → **SKIP to D11**
___ Yes, located off site-greater than 4 blocks away → **SKIP to D11**
___ No

D10. How does the primary care physician **typically** communicate with a Mental Health Specialist? (Please check one)

___ Communication does not occur
___ By telephone
___ By letter
___ Referral forms
___ Other (please specify: ______________________________)

D11. If a patient is referred for depression care, **how often** does the patient's primary care physician typically communicate with a Mental Health Specialist (MHS) about the patient's depression treatment?

___ Never   ___ Rarely   ___ Sometimes   ___ Often   ___ Always

D12. How do you find out whether the patient actually keeps his/her appointment with the Mental Health Specialist?

___ Physician or office staff call the Mental Health Specialist
___ Physician or office staff asks patient
___ Other (please specify:______________________________)
___ Physician or office staff does not follow up with MHS

D13. The following are common strategies offices might use to improve depression care. Please indicate which of the following strategies are currently used in your office. For those that are NOT used, please rate how effective they might be in improving the way depression care is delivered in your office.

	YES	NO		Definitely	Probably	Maybe	No
a. Designate an office employee to follow- up on newly diagnosed patients with depression………	_____	_____	If No,	1	2	3	4
b. Develop an information tool for providers to better monitor the care of their depressed patients .	_____	_____	If No,	1	2	3	4
c. Improve depression screening in the office…….	_____	_____	If No,	1	2	3	4
d. Identify a mental health provider who can see patients in the office……………………………………	_____	_____	If No,	1	2	3	4
e. Pay providers bonuses for improving quality of depression care………………………………………...	_____	_____	If No,	1	2	3	4
f. Identify a mental health provider to whom depressed patients can be referred……………………..	_____	_____	If No,	1	2	3	4
g. Developing linkages to faith- based resources……	_____	_____	If No,	1	2	3	4
h. Other (specify)………………………………………. .	_____	_____	If No,	1	2	3	4

E. Barriers

We want to learn more about barriers providers encounter when treating patients in your office.

E1. What are some of the most serious health problems (including mental and physical conditions) facing patients in your office?

E2. What health issues or problems that you encounter in your office do you feel you need the most help with or information on?

E3. What are some of the most significant barriers your office faces in managing your patients with **depression** (e.g., inadequate resources for screening, referral follow-up, medication access, psychosocial care)?

E4. What resources or services would be most helpful to your office in addressing the barriers to adequate **depression** care for your patients (e.g., improve screening, referral follow-up, medication access, psychosocial care)?

F. Other Practice Issues

F1. What are the most frequent **ethical** concerns that arise for you, your colleagues, and your patients in your office?

F2. How are these ethical concerns currently addressed in your office? Who addresses them?

F3. What resources or services would be most helpful to your office in addressing the sort of ethical concerns that you have described?

GENERAL NOTES

F4. Have any patients ever participated in any research studies, through the actions of your office?
__No (**Go to Question F5**)
__Yes
__Don't Know

F4a. if YES to Question **F4,** which types of research did your patients participate in?
__ Clinical Trial (drug, device, procedure; Phase I-IV)
__ Research on therapeutic or preventive interventions (i.e., treatments) excluding clinical trials
__Epidemiological, survey-based, or other observational study
__ Don't Know
__Other (specify:_____________________)

F5. Does your office subscribe to (and/or pay for) any medical journals (e.g., JAMA®, Lancet®) for your providers?
__No __Yes (specify titles:____)

F6. Does your office pay for Internet connections, and/or specialized software (e.g., Epocrates®; MDConsult®; UpToDate®, Health Sciences Library System), for your providers?
__No __Yes (specify programs:____)

F7. Does your office purchase or get consumer health information materials for distribution to patients?
__No __Yes (please give examples:____)

G. Additional Information

G1. What is your current job title? ____

G2. How long have you worked in your current position? __ __ years

G3. What is your gender? __Female __Male

G4. What is your age? __18-34 __35-44 __45-54 __55-64 __65-or older

G5. What is your race/ethnicity? __African-American __White __East Indian __Asian __Latino __Other

GENERAL NOTES ____

____

____

____

____

Thank you for participating!
